# 

**Published:** 1830-07-01

**Authors:** 


### MEDICO-CHIRURGICAL

### REVIEW,

AND

### JOURNAL

OF

### PRACTICAL MEDICINE.

*(NEW SERIES.)*

VOLUME THIRTEEN,

[1st of APRIL, to 30th of SEPTEMBER,]

1830.

VOL. XVII. of ANALYTICAL SERIES.

EDITED BY JAMES JOHNSON, M.D.

PHYSICIAN EXTRAORDINARY TO THE KING,

&c. &c. &c.

Surgeon Genl's Office LIBRARY. 57452

LONDON:

PUBLISHED BY S. HIGHLEY, 174, FLEET STREET,

AND WEBB STREET, ST. THOMAS'S HOSPITAL,

And all other Booksellers.

WI
ME 75 T

PRINTED BY G. HAYDEN,
Little College Street, Westminster.

### CORRESPONDENCE.

The Paper of Dr. Hugh Fraser (of the Civil Hospital of Gibraltar) is received. The paper shall find a place in the next Number of this Journal.

### Index to the first 20 Volumes of this Journal.

We have received many pressing letters on the subject of a General Index, and some generous offers as to its construction. To Dr. C. of W. we return grateful thanks, and shall write to him privately. The Index may be depended upon. We shall endeavour, by small type and great labour, to make it a very trifling tax on our Subscribers.

